# Impact of structural polymorphism for the *Helicobacter pylori* CagA oncoprotein on binding to polarity-regulating kinase PAR1b

**DOI:** 10.1038/srep30031

**Published:** 2016-07-22

**Authors:** Hiroko Nishikawa, Takeru Hayashi, Fumio Arisaka, Toshiya Senda, Masanori Hatakeyama

**Affiliations:** 1 Division of Microbiology, Graduate School of Medicine, The University of Tokyo, Tokyo 113-0033, Japan; 2 CREST, Japan Science and Technology Agency, Saitama 332-0012, Japan; 3 Max Planck-The University of Tokyo Center for Integrative Inflammology, Tokyo 113-0033, Japan; 4 College of Bioresource Sciences, Nihon University, Fujisawa 252-0880, Japan; 5 Structural Biology Research Center, Institute of Materials Structure Science, High Energy Accelerator Research Organization (KEK), Tsukuba 305-0801, Japan

## Abstract

Chronic infection with *cagA*-positive *Helicobacter pylori* is the strongest risk factor for atrophic gastritis, peptic ulcers, and gastric cancer. CagA, the product of the *cagA* gene, is a bacterial oncoprotein, which, upon delivery into gastric epithelial cells, binds to and inhibits the polarity-regulating kinase, partitioning-defective 1b (PAR1b) [also known as microtubule affinity-regulating kinase 2 (MARK2)], via its CagA multimerization (CM) motif. The inhibition of PAR1b elicits junctional and polarity defects, rendering cells susceptible to oncogenesis. Notably, the polymorphism in the CM motif has been identified among geographic variants of CagA, differing in either the copy number or the sequence composition. In this study, through quantitative analysis of the complex formation between CagA and PAR1b, we found that several CagA species have acquired elevated PAR1b-binding activity via duplication of the CM motifs, while others have lost their PAR1b-binding activity. We also found that strength of CagA-PAR1b interaction was proportional to the degrees of stress fiber formation and tight junctional disruption by CagA in gastric epithelial cells. These results indicate that the CM polymorphism is a determinant for the magnitude of CagA-mediated deregulation of the cytoskeletal system and thereby possibly affects disease outcome of *cagA*-positive *H. pylori* infection, including gastric cancer.

*Helicobacter pylori* is a spiral-shaped Gram-negative bacterium that colonizes approximately 50% of the world’s population and is the causative agent of gastrointestinal diseases such as atrophic gastritis and peptic ulcers^1^,^2^. Infection with *H. pylori* is the strongest risk factor for the development of gastric cancer^3^,^4^, the third leading cause of cancer deaths worldwide^5^. Manifestations associated with chronic *H. pylori* infection vary considerably among distinct geographic regions and these differences have been attributed at least in part to polymorphisms of the *H. pylori* virulence factors such as CagA, VacA, IceA, BabA, DupA, and OipA^6^,^7^. Among those factors, much attention has been given to the structure-function relationship of CagA because of its strong association with gastric cancer^8^,^9^.

CagA, a 120~145-kDa protein, is injected into attached gastric epithelial cells via the bacterial type IV secretion system^10^,^11^. CagA comprises a structured N-terminal domain that tethers the protein to the inner leaflet of the plasma membrane^12^,^13^ and an intrinsically disordered C-terminal tail that contains variable repeats of a tyrosine phosphorylation motif called the Glu-Pro-Ile-Tyr-Ala (EPIYA) motif^14^,^15^. Upon delivery into the host cell, the EPIYA motifs are tyrosine-phosphorylated by host kinases and thereby bind promiscuously to a number of SH2 domain-containing proteins, such as SHP2 tyrosine phosphatase^16^, C-terminal Src kinase (Csk)^17^ and adaptor protein Crk^18^, to perturb their activities. Based on the amino acid sequences flanking these EPIYA motifs, four distinct EPIYA segments, EPIYA-A, -B, -C, and -D, with each segment carrying a single EPIYA motif, have been defined^19^. Approximately 60% of the *H. pylori* strains circulating all over the world, except East Asian countries such as Japan, China and Korea, produce Western CagA, a CagA species containing EPIYA-A, EPIYA-B and a variable number of EPIYA-C segments, typically present in 1–3 repeats in tandem^2^,^19^,^20^. On the other hand, almost all of the *H. pylori* strains isolated in East Asian countries produce a different class of CagA, termed the East Asian CagA, which, like Western CagA, contains the EPIYA-A and EPIYA-B segments but, instead of the EPIYA-C segments, carries a distinct segment termed EPIYA-D^2^,^19^. East Asian CagA has been linked to the high incidence of gastric cancer in East Asian countries compared to that in the rest of the world^21^. Among Western CagA species, those containing multiple EPIYA-C segments have been more closely associated with gastric cancer than those containing a single EPIYA-C segment^20^. In addition to the EPIYA tyrosine phosphorylation motif, the EPIYA-C segment also carries a 16-amino-acid sequence termed the CagA multimerization (CM) motif 22. Initially identified as a motif that mediates CagA multimerization (dimerization), it was later discovered that the motif serves as a binding site for the polarity-regulating kinase, partitioning-defective 1 (PAR1), which can exist as a multimer (most probably dimer), in a phosphorylation-independent manner^23^. The crystal structure of the complex between PAR1b (39–364) and a fragment of Western CagA containing the CM motifs has revealed that the CM motif mimics host substrates to bind to the active site of PAR1b, inhibiting its kinase activity^24^. Like EPIYA segments, polymorphism also exists in the CM motif between Western CagA and East Asian CagA^22^. Western CagA typically possesses 2–4 repeats of the Western CM (CM^W^) motif as it usually contains 1–3 tandem repeats of the EPIYA-C segment that is followed by a single CM motif immediately downstream of this repeat region. East Asian CagA, in contrast, does not contain a CM motif in its EPIYA-D segment but carries a single East Asian CM (CM^E^) motif, which differs from the CM^W^ by 5 amino acid residues, immediately downstream of the EPIYA-D segment^22^.

PAR1 is a highly conserved family of serine/threonine kinases, originally isolated in a screen for partitioning-defective mutants of *C. elegans* early embryos^25^. Independently, mammalian PAR1 was identified as microtubule affinity-regulating kinase, MARK, a family of kinases that phosphorylate microtubule-associated proteins, MAPs, such as tau, MAP2, and MAP4 at their microtubule-binding C-terminal repeats to release the MAPs from microtubules and destabilize them^26^,^27^. Mammalian PAR1 is comprised of four isoforms, PAR1a-d. The major isoform in epithelial cells, PAR1b, has been shown to localize at the basolateral membrane of polarized epithelial monolayers where it plays crucial roles in the establishment and maintenance of apical-basal epithelial cell polarity^28^,^29^,^30^. Accordingly, inhibition of PAR1b by *H. pylori* CagA in polarized epithelial monolayers provokes cell polarity and junctional defects^23^,^31^. Moreover, recent studies have indicated that the role of PAR1b in the cytoskeleton is not confined just to microtubule dynamics but also extends to filamentous actin (F-actin) rearrangements^32^,^33^. PAR1b inhibits a RhoA-specific GEF, GEF-H1, by phosphorylation to down-regulate RhoA and thereby inhibit stress fiber formation^34^. Stress fibers are contractile bundles of actomyosin anchored to focal adhesions, which generate the contractile force required for cell motility^35^,^36^.

Non-canonical CM motifs have been identified from other parts of the world such as Amerindian I CM (CM^AmI^) and Amerindian II CM (CM^AmII^) from an indigenous tribe of the Peruvian Amazon^37^,^38^,^39^,^40^. In this study, we examined for the first time the role of CM polymorphism in the CagA-PAR1b interaction through quantitative analysis and evaluated the impact of the CM polymorphism on the CagA action towards the actin cytoskeletal system to better understand the gastrointestinal pathogenesis caused by *cagA*-positive *H. pylori*.

## Results

### CM motifs are required for CagA-PAR1b interaction *in vitro*

**Figure 1:**
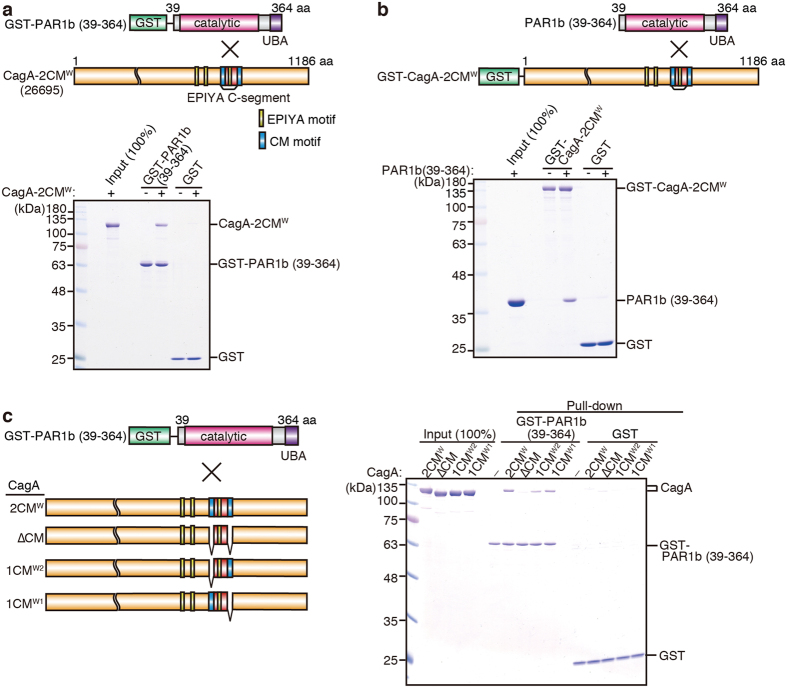
*In vitro* reconstitution of CagA-PAR1b interaction. (**a**) Schematic diagram of GST-PAR1b (39–364), which contains the catalytic and the UBA domains, and CagA-2CM^W^ (*H. pylori* 26695 strain) which contains 2 CM motifs: one in the EPIYA-C segment and the other immediately downstream of the segment (*top*). Results for the GST pull-down between GST-PAR1b (39–364) and CagA-2CM^W^ (*bottom*). (**b**) Schematic diagram of PAR1b (39–364) and GST-CagA-2CM^W^ (*top*) and the results of the GST pull-down using these constructs (*bottom*). (**c**) Schematic diagram of GST-PAR1b (39–364) and GST-CagA-2CM^W^ constructs with CM deletions (*left*) and the results of the GST pull-down using these constructs (*right*). All pull-downs were performed in HBS-P buffer. Samples were resolved on SDS-PAGE gels and visualized by CBB.

Previous studies have shown that CagA and PAR1b specifically interact in COS-7 cells and human gastric AGS cells^23^,^31^,^41^. To determine whether the interaction is direct or not, recombinant PAR1b (39–364), which contains both the catalytic and the ubiquitin-associated (UBA) domains, was expressed as a GST fusion protein in *E. coli* and then purified (Fig. 1a, top). A canonical Western ABC type CagA (CagA-2CM^W^), which contains an EPIYA-C segment and thus two CM^W^ motifs (one in the EPIYA-C segment and the other immediately downstream of EPIYA-C), was also expressed in *E. coli* and purified. A GST pull-down assay revealed that CagA-2CM^W^ bound to GST-PAR1b (39–364), while it did not bind to the GST protein alone (Fig. 1a, bottom). To ascertain that the interaction is reproducible in a reciprocal pull-down, the GST-CagA-2CM^W^ construct was used to pull-down PAR1b (39–364). As shown in Fig. 1b, GST-CagA-2CM^W^ pulled down PAR1b (39–364), while the GST control protein did not (Fig. 1b, bottom). Collectively, these results indicated that CagA-2CM^W^ interacted directly and specifically with PAR1b (39–364).

Next, to determine whether the CM motif is required for direct interaction of CagA with PAR1b *in vitro*, we generated three CagA-2CM^W^ mutants: one that has the first CM (CagA-1CM^W1^), another that only has the second CM (CagA-1CM^W2^), and lastly, one that has no CM motif (CagA-ΔCM) (Fig. 1c, left). The recombinant proteins obtained were then mixed with GST-PAR1b (39–364) and a GST pull-down assay was performed. In accordance with our previous studies using cultured cells, GST-PAR1b (39–364) bound to CagA-2CM^W^ but not to CagA-ΔCM^23^,^31^ (Fig. 1c, right). The binding of GST-PAR1b (39–364) with CagA-2CM^W^ was stronger than that with CagA-1CM^W2^ or CagA-1CM^W1^, carrying only a single CM motif. We also observed a CM-dependent interaction between CagA and full-length PAR1b (Supplementary Fig. S1). Thus, at least a single CM motif was required for direct interaction of CagA with PAR1b.

### The two CM motifs in the C-terminus of CagA are not mutually exclusive in PAR1b binding

**Figure 2:**
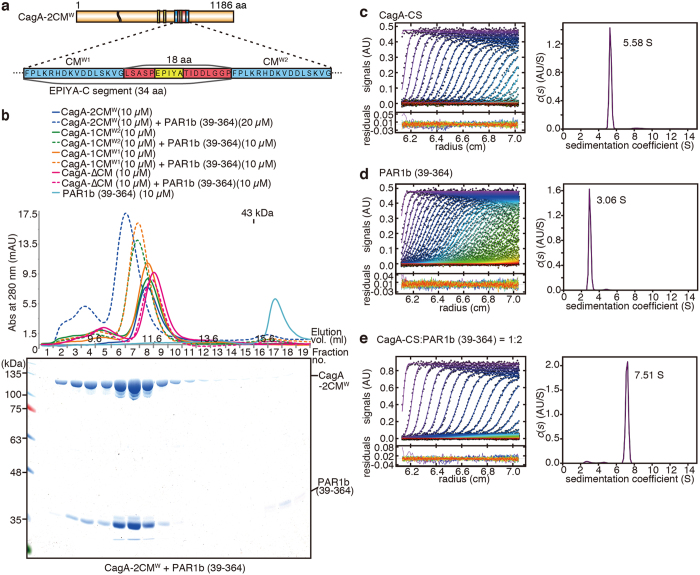
Two PAR1b can simultaneously bind to CagA. (**a**) Schematic diagram of CagA-2CM^W^ and a blow-up of the region containing the CM motifs. CM^W1^ resides in the 34-amino-acid EPIYA-C segment and CM^W2^ flanks the segment on the C-terminal side. The two CM sequences are 18 amino acid residues apart. (**b**) Results from the size-exclusion chromatography of CagA-PAR1b (39–364) complexes analysed on Superdex 200 10/300 GL using HBS-P as running buffer (*top*). Fractionated CagA-2CM + PAR1b (39–364) complex was further resolved on SDS-PAGE gel and stained by CBB (*bottom*). (**c–e**) Analytical ultracentrifugation of 5.5 μM CagA-C1164S (CagA-CS) (**c**), 11 μM PAR1b (39–364) (**d**), and 5.5 μM CagA-CS + 11 μM PAR1b (39–364) (**e**). Raw absorbance distributions, the best-fit model (*left*), and the continuous sedimentation distributions *c(s*) (*right*) calculated by the SEDFIT program.

Co-crystallization of the CagA peptide and PAR1b (39–364) has shown that the bound form of PAR1b (39–364) is approximately 40 Å in width^24^. Since the two CM motifs in CagA-2CM^W^ reside in the intrinsically disordered region of CagA^13^ and are 18 amino acid residues apart, or approximately 60 Å in the extended form, this region should be able to accommodate the binding of two PAR1b molecules (Fig. 2a). However, many intrinsically disordered proteins can induce folding upon binding to their target^42^. We therefore speculated that the binding of PAR1b (39–364) to one of the two CM motifs in CagA-2CM^W^ might induce folding of the surrounding regions and inhibit the binding of PAR1b to the other CM motif. Thus, to determine whether the two CM motifs of CagA-2CM^W^ can simultaneously bind to PAR1b or not, recombinant CagA-2CM^W^ proteins with differential CM motif mutations were mixed *in vitro* with recombinant PAR1b (39–364) and analysed by size-exclusion chromatography (SEC) (Fig. 2b, top). When CagA-ΔCM, CagA-1CM^W2^, CagA-1CM^W1^, and CagA-2CM^W^ were individually analyzed by SEC, the major peaks were very close to each other, eluting at 11.6 ml, 11.4 ml, 11.4 ml, and 11.4 ml, respectively. It should be noted that these major peaks represent monomeric CagA and the sub-peaks that elute earlier are due to artificial dimerization of CagA, caused *in vitro* via a C-terminal cysteine residue, which can oxidize to form disulphide bonds^13^. On the other hand, the major peak of PAR1b (39–364) was at 15.9 ml and was smaller than the 43-kDa marker. When CagA-ΔCM was mixed with PAR1b (39–364) at a molar ratio of 1:1, the major peak eluted in a position similar to the peak of CagA-ΔCM alone at 11.5 ml, suggesting that it does not bind to PAR1b (39–364). When CagA-1CM^W2^ or CagA-1CM^W1^ was mixed with PAR1b (39–364), major peaks for the two complexes eluted significantly earlier than peaks of CagA-1CM^W2^ and CagA-1CM^W1^ alone, at 11.0 ml. Notably, when CagA-2CM^W^ and PAR1b (39–364) were mixed at a molar ratio of 1:2, the major peak eluted markedly earlier at 10.6 ml and the PAR1b (39–364) peak was significantly reduced, suggesting that most of PAR1b (39–364) formed complexes with CagA-2CM^W^. SEC fractions of CagA-2CM^W^ and PAR1b (39–364) were then resolved on SDS-PAGE gel and stained by Coomassie Brilliant Blue (CBB) (Fig. 2b, bottom). Quantification of individual bands in fraction 7, the major peak, revealed that this peak contained CagA-2CM^W^ and PAR1b (39–364) at a molar ratio of 1:2. From these observations, we concluded that the two CM motifs in a single CagA-2CM^W^ molecule were not mutually exclusive in PAR1b binding, suggesting that two PAR1b molecules can simultaneously bind to a single CagA protein despite the proximity of the two CM motifs.

To further confirm that two PAR1b molecules can simultaneously bind to a single CagA-2CM^W^ molecule, analytical ultracentrifugation (AUC) was performed. AUC has an advantage over SEC in that it provides a more precise measurement of molecular weights, which do not depend on the molecular shape. Indeed, SEC analysis previously showed that CagA-2CM^W^, whose sequence-based molecular weight is 134 kDa^43^, eluted earlier than the 257-kDa marker, most likely due to its flat plate-like structure^13^. This time, to avoid artificial dimerization of CagA through oxidization, the oxidizable cysteine residue (C1164) of CagA-2CM^W^ was mutated to a serine residue (CagA-CS). When CagA-CS and PAR1b (39–364) were analysed individually by AUC, they produced a peak with sedimentation coefficients of 5.58 S and 3.06 S, corresponding to the molecular weights of CagA-CS (121 kDa) and PAR1b (36.8 kDa), respectively (Fig. 2c,d). Since the sequence-based molecular weight of PAR1b (39–364) is 39.4 kDa, the AUC results are consistent with the conclusion that the two recombinant proteins existed as monomers in solution. When CagA-CS and PAR1b (39–364) were mixed at a ratio of 1:2 and analysed by AUC, it produced a sharp major peak with a sedimentation coefficient of 7.51 S (93% of the total loading concentration; Fig. 2e). Conversion of *c*(*s*) to *c*(*M*) gave the molecular weight of 182 kDa which was slightly (6%) smaller than that of the expected 1:2 complex but (15%) larger than that of the 1:1 complex. In addition, the remaining monomeric molecular species of CagA-CS and PAR1b (39–364) specimens in the solution were less than 2 or 3% of the loading concentration. We therefore concluded that CagA-CS and PAR1b interact in a 1:2 stoichiometry. Thus, the results from the AUC experiment were in agreement with the results from the SEC experiment and provided additional evidence that a CagA-2CM^W^ molecule was capable of simultaneously interacting with two PAR1b molecules via duplicated CM motifs.

### Tandem CM motifs in Western CagA synergize in PAR1b binding

**Figure 3:**
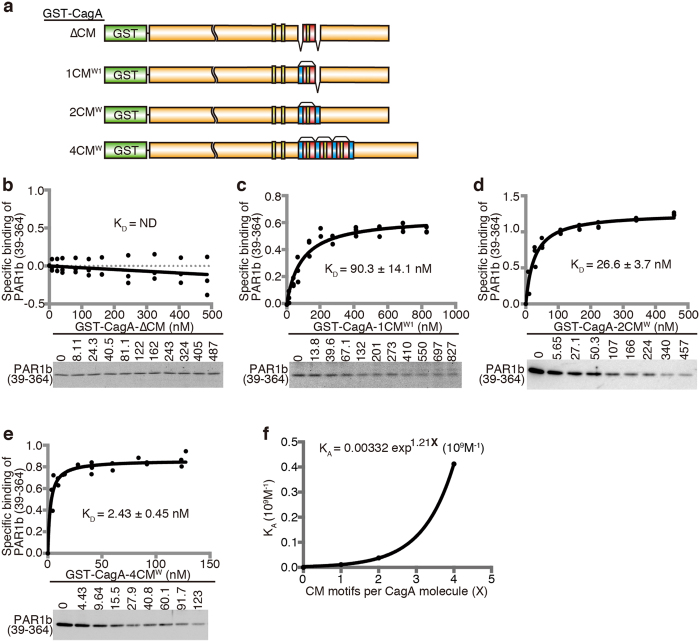
Tandem CM motifs generate strong binding affinity to PAR1b. (**a**) Schematic diagram of GST-CagA constructs used. CagA-4CM^W^ was derived from *H. pylori* NCTC11637 strain. (**b–e**) Saturation binding curves obtained by quantitative GST binding assay between PAR1b (39–364) and GST-CagA-ΔCM (**b**), GST-CagA-1CM^W1^ (**c**), GST-CagA-2CM^W^ (**d**), or GST-CagA-4CM^W^ (**e**). Values shown are dissociation constants (K_D_) ± SE, n = 3. Bands at the bottom of each graph shows a representative CBB stained gel or an immunoblot of unbound PAR1b (39–364). Full-length gels and blots are presented in Supplementary Fig. S2. (**f**) Graph and equation depicting the relationship between the number of CM motifs per molecule of CagA and their association constants (K_A_) with PAR1b (39–364).

Since clinically isolated Western CagA species generally carry 2–4 copies of the CM^W^ motif, which are not mutually exclusive to each other in terms of binding to PAR1b, we investigated whether carrying multiple repeats of CM^W^ motifs in close proximity confers an advantage in binding to PAR1b by directly comparing the binding affinities of CagA constructs with different numbers of CM^W^ motifs to PAR1b (39–364). To obtain quantitative measurements of the binding affinities between the CagA constructs and PAR1b (39–364), we used the quantitative GST pull-down assay^44^. To this end, a GST tag was fused to CagA containing 0–4 repeats of the CM^W^ motifs (Fig. 3a). The resulting GST-CagA fusion proteins were bound to beads and aliquoted to each reaction tube in a wide range of concentrations and then mixed with a fixed low concentration of PAR1b (39–364). Unbound PAR1b (39–364) was quantified after running the supernatant of the reaction on an SDS-PAGE gel. Bound PAR1b (39–364) was calculated by subtracting the unbound PAR1b (39–364) from total PAR1b (39–364) and then a saturation binding curve was obtained to determine the apparent dissociation constant (K_D_) (Fig. 3b–e). The K_D_ value for CagA-ΔCM could not be determined as no significant binding was observed (Fig. 3b), in accordance with the results shown in Figs 1c and 2b. The binding affinity, as determined by the K_D_ value, strengthened in correlation with the number of tandem CM^W^ motifs. CagA-1CM^W1^ and CagA-2CM^W^ exhibited K_D_ values of 90.3 ± 14.1 nM and 26.6 ± 3.7 nM, respectively (Fig. 3c,d). Strikingly, CagA-4CM^W^, containing four CM^W^ motifs, displayed a marked increase in PAR1b-binding affinity (K_D_ = 2.43 ± 0.45 nM), which was more than 10-fold greater than that of CagA-2CM^W^ and more than 37-fold greater than that of CagA-1CM^W1^.

To further investigate the relationship between the number of CM motifs per CagA molecule and the binding affinity of the CagA-PAR1b interaction, the apparent association constant (K_A_ = 1/K_D_) was plotted against the number of tandem CM motifs (Fig. 3f). If the CM motifs bind to PAR1b (39–364) independently of each other and the K_A_ value of CagA-PAR1b increases only as a result of the increase in the concentration of CM motifs, then the K_A_ value should increase proportionally to the number of CM motifs per CagA molecule. However, this was clearly not the case; an exponential curve of K_A_ = 0.00332exp^1.21X^ 10^9^M^−1^ (where X is the number of CM motifs) was obtained by curve fitting. The result therefore indicated that the tandem CM motifs in a CagA molecule act synergistically in generating strong PAR1b binding, which would otherwise require a very high concentration of intracellular CagA if only a single CM motif existed per CagA molecule.

### Binding affinities of geographic CM variations to PAR1b

While Western CagA species are known to contain multiple (usually 2–4) copies of the CM^W^ motif, East Asian CagA species contain only a single copy of the CM^E^ motif, which differs by 5 amino acids from its Western counterpart^22^. Interestingly, this sequence alteration seems to be crucial for the CM^E^ motif in generating a stronger binding affinity to PAR1b than the CM^W^ motif 31. Additional variations in the CM motif have also been discovered from other parts of the world^37^,^38^,^40^. They include CM^AmI^ and CM^AmII^ motifs from Amerindian natives of the Peruvian Amazon^37^,^39^.

**Figure 4:**
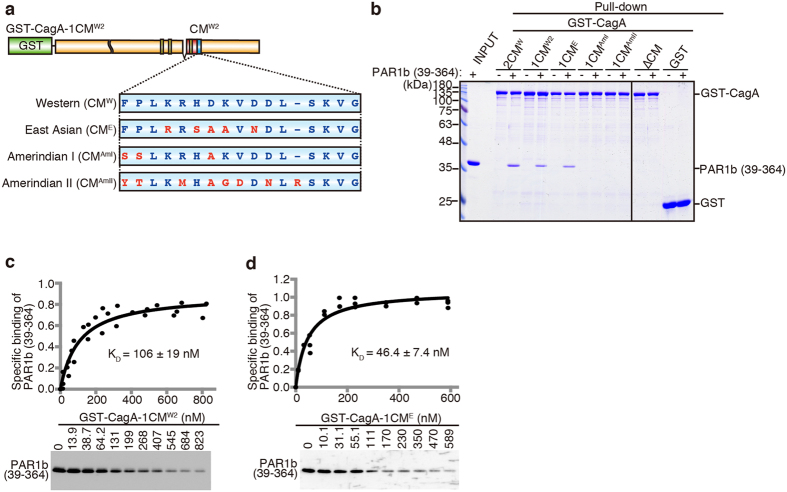
Binding affinities of regional CagA CM variations to PAR1b. (**a**) Schematic diagram of GST-CagA-1CM^W2^ carrying various regional CM motifs at the CM^W2^ position. (**b**) GST pull-down between various GST-CagA-1CM^X^ constructs with various CM motifs and PAR1b (39–364). (**c,d**) Saturation binding curves obtained by the quantitative GST binding assay between PAR1b (39–364) and GST-CagA-1CM^W2^ (**c**) or GST-CagA-1CM^E^ (**d**). Values are dissociation constants (K_D_) ± SE, n = 3. Full-length gels and blots are presented in Supplementary Fig. S3.

To compare the PAR1b binding activities of these geographic CM variants with the binding activity of CM^W^, we made use of the approach used for determining PAR1b binding of CagA-2CM^W^ (Fig. 1c). Since CM^E^ and CM^AmII^ motifs usually exist as a single copy at the CM^W2^ position of their respective geographic CagA variants, we generated chimeric GST-CagA constructs by replacing the CM^W2^ motif of GST-CagA-1CM^W2^ with that of the various geographic CM variants (GST-CagA-1CM^X^; X as the variant) (Fig. 4a). A GST pull-down assay showed that GST-CagA-1CM^E^ bound to PAR1b (39–364) as previously described^31^ (Fig. 4b). In contrast, neither GST-CagA-1CM^AmI^ nor GST-CagA-1CM^AmII^ bound to PAR1b (39–364).

For a more quantitative evaluation of the binding affinities of CM^W^ and CM^E^ motifs to PAR1b, we performed quantitative GST pull-down assays using the chimeric GST-CagA proteins to determine the K_D_ values. As shown in Fig. 4c, the K_D_ value between GST-CagA-1CM^W2^, which contains a single CM^W^ motif, and PAR1b (39–364) was 106 ± 19 nM, whereas the K_D_ value between GST-CagA-1CM^E^, which contains a single CM^E^ motif, and PAR1b (39–364) was 46.4 ± 7.4 nM. Hence, the binding affinity of the CM^E^ motif to PAR1b (39–364) was twice as strong as that of the CM^W^ motif, being consistent with the results obtained from co-immunoprecipitation assays using a COS-7 overexpression system which showed that the binding activity of a single CM^E^ motif is equivalent to that of two tandem CM^W^ motifs^31^. K_D_ values determined in this study are summarized in Table 1. It showed that the binding affinity of the CM^W^ motif to PAR1b was constant irrespective of its position at CM^W1^ or CM^W2^ in CagA. Table 1 also showed that while the binding affinity of CagA containing a single CM^E^ motif was twice as strong as that of CagA containing a single CM^W^ motif, duplication of the CM^W^ motif in the context of CagA potentiated the PAR1b-binding affinity of CagA to a level comparable to that of CagA possessing a single CM^E^ motif.

### Augmentation of CagA-PAR1b binding potentiates stress fiber formation in cells

**Figure 5:**
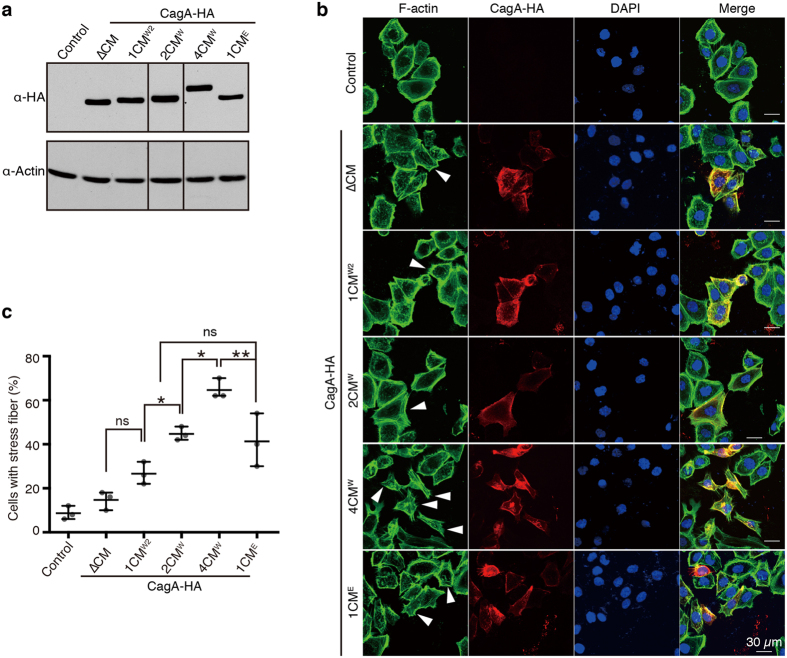
CM-dependent augmentation of stress fiber formation by CagA. (**a**) Immunoblots of AGS total cell lysates 24 h after transfection with the various pCDH-EF1-CagA-HA expression vectors as indicated. CagA-HA was detected by using anti-HA (3F10) as primary antibody. Control indicates cells transfected with the empty pCDH-EF1 vector. Full-length blot is presented in Supplementary Fig. S4. (**b**) Representative fluorescent images of AGS cells after transfection. F-actin was visualized by Alexa Fluor 488 phalloidin. CagA-HA was detected using anti-HA (C29F4) followed by Alexa Fluor 546 anti-rabbit antibodies. Nuclei were stained with DAPI. Arrowheads mark CagA-HA expressing cells positive for stress fiber. (**c**) Quantification of CagA-HA expressing AGS cells displaying stress fiber. 50 CagA-positive cells per construct were analysed for stress fiber formation per experiment. For control cells, 50% of cells in each image were chosen randomly and assessed for stress fiber formation. Error bars represent mean ± range, n = 3. *P < 0.05, **P < 0.01, repeated measures one-way ANOVA, post-hoc Tukey’s test.

Western CagA species was capable of undergoing strong PAR1b binding through tandem duplication of the CM^W^ motif. Also, East Asian CagA carrying a single CM^E^ motif exhibited a PAR1b-binding affinity similar to that of Western CagA carrying two CM^W^ motifs *in vitro*. However, it was possible that, in cells, CagA with a single CM^W^ motif would be sufficient in saturating endogenous PAR1b binding. Moreover, the EPIYA-C motif, which serves as an SHP2-binding site upon tyrosine phosphorylation, exists in the 18-amino-acid spacer between the tandem CM^W^ motifs, and the CagA-SHP2 interaction could competitively inhibit CagA-PAR1b interaction in a cellular context. Given this, we decided to assess the biological impact of different CM polymorphisms with regard to the cytoskeletal role of PAR1b. To this end, we examined the effect of CagA on the formation of stress fibers in AGS human gastric epithelial cells. Stress fibers are contractile bundles of actomyosin that play an important role in cell adhesion, cell motility, and morphogenesis^35^,^36^. We previously showed that PAR1b regulates stress fiber formation by suppressing RhoA activity via inhibition of a Rho-specific GEF, GEF-H1^34^. If CagA with more CM motifs inhibits endogenous PAR1b more efficiently than does CagA with fewer CM motifs through enhanced complex formation, then cultured cells expressing CagA species with more CM motifs should display greater stress fiber formation. Thus, HA-tagged constructs of CagA-ΔCM, CagA-1CM^W2^, CagA-2CM^W^, CagA-4CM^W^, and CagA-1CM^E^ were transiently expressed in AGS cells. The cells were then immunostained with an anti-HA antibody to identify CagA-HA-expressing cells (Fig. 5a,b). F-actin was visualized by fluorescent-labelled phalloidin, and the magnitude of stress fiber formation was quantitatively assessed. CagA was localized to the membrane as previously described^16^,^45^. The majority of AGS cells expressing CagA-ΔCM displayed strong cortical staining of F-actin, similar to the control cells, while only 15 ± 5% of CagA-positive cells exhibited stress fiber formation (Fig. 5c). Cells expressing CagA with a single copy of the CM motif, CagA-1CM^W2^, did not show a significant increase (*P* = 0.157) in the proportion of cells with stress fibers (27 ± 5%) compared to cells expressing CagA-ΔCM (15 ± 5%). However a significant increase in the proportion of cells displaying stress fiber formation was observed in cells expressing CagA-2CM^W^ (45 ± 3%; *P* = 0.020 compared to CagA-1CM^W2^) and a further increase was observed in cells expressing CagA-4CM^W^ (65 ± 5%; *P* = 0.010 compared to CagA-2CM^W^). Of note, the morphological change observed in AGS cells expressing CagA-4CM^W^ was attributable to SHP2 deregulation by CagA, the activity of which is proportional to the number of EPIYA-C segments^46^. While we cannot exclude the potential contribution of EPIYA-C segments to stress fiber formation in cells expressing CagA-4CM^W^, the other three constructs, CagA-ΔCM, CagA-1CM^W2^ and CagA-2CM^W^, all contain only a single EPIYA-C motif and thus can be compared directly. Tandem duplication of CM motifs in CagA molecules may therefore increase the proportion of cells displaying stress fiber formation, which reflects the degree of CagA binding to PAR1b in cells. Furthermore, when the chimeric CagA-1CM^E^, which carries a single CM^E^ motif, was transiently expressed in AGS cells, the number of cells displaying stress fiber formation was similar to that of cells expressing CagA-2CM^W^ (Fig. 5c; *P* = 0.970), also being consistent with our *in vitro* results showing that CagA with two CM^W^ motifs is similar to CagA with one CM^E^ motif in terms of PAR1b binding (Table 1).

### Inverse correlation between the number of CagA CM motifs and transepithelial electrical resistance (TER)

**Figure 6:**
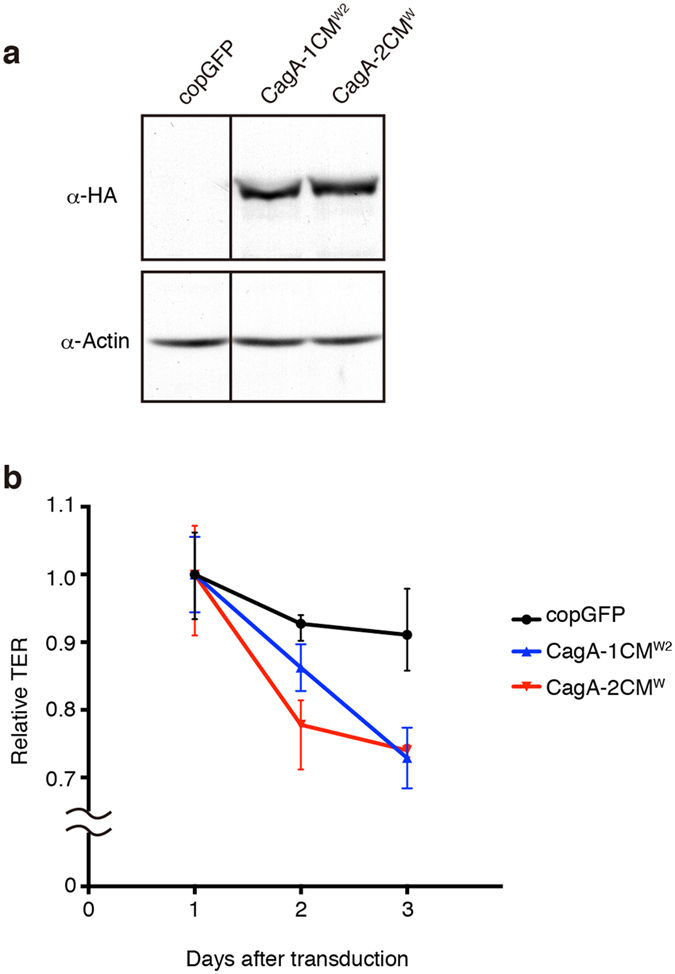
CM-dependent disruption of polarized MDCK monolayers. (**a**) Immunoblots of MDCK total cell lysates 3 days after transduction with the lentiviral constructs as indicated. CagA-HA was detected by using anti-HA (6E2) as primary antibody. The copGFP-transducing lentivirus was used as a negative control. Full-length blot is presented in Supplementary Fig. S5. (**b**) Relative TER of polarized MDCK monolayers normalized to Day 1 post transduction. Error bars represent mean ± range, n = 3.

Finally, we decided to investigate the effects of the series of CagA CM mutants on cell-cell interaction by measuring transepithelial electrical resistance (TER), which reflects the strength of the tight junction, using polarized Madin-Darby canine kidney (MDCK) epithelial monolayers. The experiment makes it possible to provide objective data for quantitatively evaluating the degree by which each CagA mutant perturbs tight junction integrity. Previous application of TER showed that, when expressed in polarized MDCK monolayers, CagA-1CM^E^ disrupts the tight junction more than CagA-1CM^W2 ^31. To address whether the disruption of the tight junctions is proportional to the number of CM^W^ motifs, we infected polarized MDCK cells with lentivirus transducing copGFP (negative control), CagA-1CM^W2^, or CagA-2CM^W^ and measured TER across the polarized MDCK monolayer for 3 days (Fig. 6a,b). Two days after transduction, CagA-2CM^W^ elicited a sharp decrease in TER compared to control, and the effect seemed reach a saturation point at Day 3. CagA-1CM^W2^, on the other hand, disrupted the tight junctions more slowly than CagA-2CM^W^. Thus, the results of the experiment revealed that the degree of CagA-mediated disruption of tight junctions was proportional to the number of CM motifs, which determines the binding strength of CagA to PAR1b and subsequent inhibition of PAR1b kinase activity.

## Discussion

Structural polymorphisms in the C-terminal tail of CagA, which correlate with the differential geographical distribution of *H. pylori*, have been implicated to influence the development of gastrointestinal diseases^8^,^9^. In this study, we investigated the CM motif by using quantitative methods to address how the structural polymorphism of this motif influences the PAR1b-binding activity of CagA. We found that in the case of Western CagA, an increase in the number of CM motifs in a CagA molecule augments its binding affinity to PAR1b in an exponential manner (Fig. 3f). Strikingly, CagA-4CM^W^ generated a K_D_ value that is more than 37-fold higher than that of CagA-1CM^W1^. This robust augmentation of the CagA-PAR1b interaction can be explained by the fact that when the first CM motif of a CagA molecule interacts with a PAR1b molecule, it brings the other CM motifs to the vicinity of the PAR1b molecule. Upon dissociation of PAR1b from the first CM motif, the other CM motifs can immediately recapture PAR1b. Thus, an increase in the CM copy number would increase the chances of recapturing PAR1b on the same CagA molecule, potentiating the overall binding affinity of the CagA-PAR1b interaction.

Intriguingly, a single CM^E^ motif can generate a binding affinity to PAR1b that is similar to that of two tandem CM^W^ motifs (Fig. 3d and 4d). Hence *H. pylori* has evolved two different strategies for efficiently inhibiting endogenous PAR1b. The first strategy is quantitative, by increasing the number of tandem CM motifs, as is the case with Western CagA, and the second strategy is qualitative, by base substitution within the CM motif, as is the case with East Asian CagA. We also showed that neither CM^AmI^ nor CM^AmII^ motifs bound to PAR1b, confirming the results of a previous co-immunoprecipitation study showing that the binding of PAR1b to the Amerindian CagA proteins is extremely weak, if any^39^.

PAR1b is a master regulator for the establishment and maintenance of polarity^30^. Inhibition of this kinase by CagA has been shown to cause disruption of the tight junctions, loss of polarity^23^, microtubule disruption^32^, and promotion of transcytosis^47^. The dominant prevalence of East Asian CagA and Western CagA subtypes in much of the world suggests that either a single copy of CM^E^ or two CM^W^ motifs enables optimal inhibition of PAR1b, which contributes to successful colonization of *H. pylori. H. pylori* lives in a surprisingly nutrient-deprived environment, and iron uptake from the host has been shown to be a critical factor for the success of *H. pylori* colonization, as iron in the host’s stomach is not in its usable form^47^,^48^. The bacterium therefore disrupts host cell polarity, most likely through PAR1b inhibition, to promote transcytosis of iron from the basolateral side of the host cell^47^. Interestingly, *H. pylori* harvested from an iron-depleted Mongolian gerbil model has been reported to exhibit enhanced virulence and to deliver elevated levels of CagA into host cells, presumably to obtain iron by promoting more transcytosis^49^. While in normal circumstances, Western *H. pylori* expressing CagA-ABC, which contains two CM^W^ sequences, may prevail in the stomach due to higher resistance to low pH^20^, selective pressures in a more nutrient-deprived environment may give substantial advantage to *H. pylori* strains that express CagA with larger numbers of CM motifs, in a mixed population. In such a case, if the C-terminal tail were structured, the protein would have to be larger to accommodate for multiple binding sites, making it more costly and disadvantageous for *H. pylori* to produce CagA with limited resources^50^,^51^. Thus, from *H. pylori*’s point of view, duplication of CM motifs in an intrinsically disordered region may offer a cost-effective solution to produce a potent effector protein. On a similar note, the Amerindian *H. pylori* strains have been outcompeted considerably by Western strains in the Latin American Mestizo and urban Amerindian populations, which has been attributed to their reduced fitness compared to Western strains^37^,^38^,^39^,^52^. Part of this reduced fitness may arise from the inability of Amerindian CagA to bind PAR1b as shown in this study.

The evolution of virulence factors in pathogens is influenced by a balance between the costs and benefits of virulence. Depending on the size of host populations and the degree of pathological impacts, pathogens may evolve toward either increasing or decreasing virulence because the death or serious disability of the host substantially influences the expansion of pathogens. Loss of PAR1b-binding activity in CagA indicates that such a CagA variant only weakly induces mucosal damage and thereby ameliorates immune/inflammatory responses, which enable long-term *H. pylori* colonization. At the same time, however, reduced virulence restrains infectivity of *H. pylori* and subsequent disease development. Thus, only in a particular society such as small and extremely isolated Amazonian rainforest tribes^37^,^38^,^40^, attenuation of CagA virulence could become an affordable option in preventing *H. pylori* from extinction.

The *in vitro* mixing experiment carried out in this study revealed successful interaction of a CagA monomer (ABC-type Western CagA possessing two CM motifs) with two PAR1 molecules simultaneously. We previously reported that CagA can dimerize in the host cells it has been delivered to^22^,^53^. Mechanistically, this CagA dimerization is mediated by the interaction of CagA with PAR1b, which is present as a dimer, via the CM motif 23,24,31,53. Since the recombinant PAR1b fragment (residues 39–364 of full-length PAR1b, which comprises 788 amino acid residues) used in this study was a monomer in solution, PAR1b dimerization may be mediated via the N-terminal or C-terminal region, which is lacking in the PAR1b fragment. Alternatively, dimerization of PAR1b might require post-translational modification or another cellular protein(s) in the mammalian host cells. In any case, our results indicate that a single CagA containing two or more CM motifs can accommodate multiple PAR1 molecules, either monomeric or dimeric forms, and thereby collectively inhibits their kinase activity.

Here, we have shown for the first time that CagA can induce stress fiber formation in a CM-dependent manner presumably through activating RhoA. Furthermore, the degree of stress fiber formation in cultured cells was in accordance with the ability of the CM polymorphism to bind PAR1b *in vitro*. Formation of stress fibers involves remodeling of actin cortical bundles into contractile actin fibers, and anchoring of these contractile fibers to focal adhesion for mechanotransduction, which may facilitate cell migration. Moreover, we showed that when CagA is transduced in polarized MDCK monolayers, the number of CM affects the acuteness in which the tight junction is disrupted. Therefore, in conjunction with the loss of tight junctions and cell polarity, stress fibers may contribute to oncogenesis by promoting invasion of surrounding tissue and metastatic dissemination^54^. It has been reported that expression of constitutively active RhoA in Madin-Darby kidney (MDCK) cells enhances stress fiber formation and causes cell morphological changes, resulting in gaps in a monolayer^55^. Thus, strong inhibition of PAR1b by CagA may compromise the integrity of the gastric epithelial layer via two mechanisms: (1) disruption of tight junctions by causing polarity defects and (2) actin remodeling via RhoA activation, thereby resulting in severe gastritis and peptic ulcers^23^,^34^. A recent study has shown that tandem duplication of the EPIYA-C segment robustly enhances SHP2-binding affinity of CagA^46^. Accordingly, strengthened binding activities to both PAR1b and SHP2 conceivably contribute to the greater incidence of gastric cancer associated with infection with *H. pylori* strains producing CagA with multiple EPIYA-C segments.

In conclusion, the degree of pathophysiological actions of *H. pylori* CagA is influenced by the sequence polymorphism of the CagA CM motif. The CM diversity may therefore contribute to the different disease manifestations caused by *cagA*-positive *H. pylori* infection. The present work will help in understanding the molecular mechanisms underlying clinical outcomes of *H. pylori* infection. Its multifaceted functions make PAR1b an attractive target for *H. pylori* in manipulating the cell host for successful colonization of the stomach, while provoking adverse and pathological effects for the host in the long term.

## Methods

### Bacterial expression vectors

pGEX-6P-2 (GE Healthcare) expression vectors for the expression of GST-CagA-2CM^W^ from *H. pylori* 26695 strain and GST-CagA-4CM^W^ from NCTC11637 strain have been described previously^12^,^13^. CM deletions and mutations were introduced by site-directed mutagenesis. In addition to a GST tag on their N-termini, all bacterial constructs used in this study contain a 6xHis tag on the C-termini for purification purposes. GST-PAR1b (39–364) and GST-PAR1b (39–364)-FLAG expression vectors were made by fusing a 6xHis tag sequence or a FLAG-6xHis tag sequence, respectively, to the 3′ end of the *PAR1b* (*39–364*) fragment by PCR and then inserting these fragments into the pGEX-6P-3 (GE Healthcare) expression vector. Human *PAR1b* (GenBank: NM_017490.3) was used as template for PCR.

### Antibodies

Anti-FLAG monoclonal antibody (M2, Sigma), anti-HA monoclonal antibodies (3F10, Roche; 6E2, Cell Signaling Technology) and anti-actin polyclonal antibody (C-11, Santa Cruz Biotechnology) were used as primary antibodies for immunoblotting. Anti-HA (C29F4, Cell Signaling Technology) was used as primary antibody for immunostaining. Horseradish peroxidase-linked anti-mouse (GE Healthcare), anti-rat (GE Healthcare) and anti-goat (Santa Cruz Biotechnology) antibodies were used as secondary antibodies in immunoblotting. Alexa Fluor 546 anti-rabbit antibody (Invitrogen) was used in immunostaining as secondary antibody.

### Protein expression and purification

Protein expression and purification of CagA and the GST control proteins were performed as previously described^13^. For the expression and purification of PAR1b (39–364) and PAR1b (39–364)-FLAG, *E. coli* BL21 harboring these expression vectors were grown at 37 °C in LB broth supplemented with ampicillin until OD_600_ reached 0.4–0.6. Protein expression was induced with 25 μM IPTG for 24 h at 18 °C, after which cells were washed and pelleted. Cells were lysed by sonication in 50 mM HEPES, 300 mM NaCl, 5% glycerol, 10 mM imidazole (adjusted to pH7.2 with NaOH) and centrifuged for 15 min at 15,000 × *g* to clear the lysate of cell debris. The cleared lysate was then mixed with Ni-NTA Agarose beads (Qiagen), and the beads were washed with 50 mM HEPES, 300 mM NaCl, 5% glycerol, 20 mM imidazole (adjusted to pH7.2 with NaOH). The protein was eluted with 50 mM HEPES, 300 mM NaCl, 5% glycerol, 250 mM imidazole (adjusted to pH7.2 with HCl). 2 mM DTT was added to the eluate, followed by mixing with Glutathione Sepharose 4B beads (GE Healthcare). Beads were washed with GST-binding buffer (50 mM Tris-Cl, pH7.3, 150 mM NaCl, 5 mM EDTA, 2 mM DTT) and the GST tag was subsequently cleaved-off by PreScission Protease (GE Healthcare) in 50 mM Tris-Cl, pH7.0, 150 mM NaCl, 1 mM EDTA, 1 mM DTT overnight. All PAR1b (39–364) purification steps were performed either on ice or at 4 °C.

### GST pull-down assay

GST-fused proteins were bound to Glutathione Sepharose 4B beads as described above, and mixed for 1 h at 4 °C with purified proteins in HBS-P buffer (10 mM HEPES-NaOH, pH 7.5, 150 mM NaCl, 0.05% Tween-20) or GST-binding buffer + 0.01% Triton X-100. Beads were then washed five times with the binding buffer, resolved on SDS-PAGE gel and stained with Coomassie Brilliant Blue (CBB) for visualization.

### Size-exclusion chromatography (SEC)

Protein samples were subjected to analysis by Superdex 200 10/300 GL filtration column (GE Healthcare) on an AKTA avant 25 system (GE Healthcare) at 4 °C using HBS-P as running buffer, at a flow rate of 0.5 ml/min. Fractionated samples were resolved by SDS-PAGE, stained with CBB and quantified using the LAS-4000 Luminescent Image Analyzer System (Fujifilm).

### Analytical ultracentrifugation (AUC)

Protein samples were dialysed against 10 mM HEPES pH 7.7, 150 mM NaCl, 1 mM EDTA and the concentration of the sample was adjusted by using the dialysate, which was also used as a reference solution. Sedimentation velocity experiments were carried out at 20 °C using a ProteomeLab XL-A analytical ultracentrifuge (Beckman Coulter). Concentration gradients were measured with UV absorption at 280 nm with no time interval. The partial specific volumes of the proteins, the buffer density and viscosity were calculated by Sednterp^56^. The distribution functions of sedimentation coefficients, *c*(*s*), were calculated using the SEDFIT program^57^, assuming that the frictional ratio was common to all the molecular species. *c*(*s*) was converted to *c*(*M*), the distribution of the molecular weights, based on the Svedberg equation, which was implemented in SEDFIT.

### Quantitative GST pull-down assay

Quantitative GST pull-down assay was designed as previously described^44^. In brief, to create a range of GST-CagA concentrations, different amounts of GST-CagA or GST bound Glutathione Sepharose 4B beads were aliquoted into reaction tubes. Unbound beads were then added to each tube accordingly, so that the total volume of beads per tube would be equal. Beads were mixed with PAR1b (39–364)-FLAG in GST-binding buffer containing 0.01% Triton-X for 20 min at 4 °C and pelleted in a standard microcentrifuge at 12,000 rpm for 2 min at 4 °C. Instead of quantifying the pellet bound fraction, which would require washing of the beads that would disturb the equilibrium of the interacting proteins, the unbound PAR1b (39–364)-FLAG was quantified by analysing the supernatant of the reaction by SDS-PAGE, followed by CBB staining or immunoblotting and quantification on a LAS-4000 Luminescent Image Analyzer System (Fujifilm). Bound PAR1b (39–364)-FLAG was determined by subtracting the unbound PAR1b (39–364)-FLAG from the total. Non-specific binding to GST-control was subtracted from bound PAR1b (39–364)-FLAG to determine the specific binding. The concentrations of GST-CagA and GST were determined by running a small sample of beads on an SDS-PAGE gel against known protein standards and quantifying the CBB-stained bands. K_D_ values were calculated by non-regression analysis using the GraphPad Prism 6 software (GraphPad Software). Non-linear regression analysis of the relationship between CM number and K_A_ values were also performed using the GraphPad Prism 6 software.

### Mammalian expression vectors

The pCDH-EF1 vector was made by excising the CMV promoter and the puromycin resistance gene from the CD510B-1 lentivector (System Biosciences) and introducing an MCS downstream of the EF-1 promoter so that the inserted gene would be expressed under the control of the EF-1 promoter. pCDH-EF1-CagA-ΔCM, -2CM^W^ and -4CM^W^ were generated by inserting chemically synthesized humanized *cagA* constructs into pCDH-EF1. Humanized *cagA-1CM*^*W2*^ was generated by deleting the first CM motif from humanized *cagA-2CM*^*W*^ by site-directed mutagenesis. *cagA-1CM*^*E*^ was also generated by site-directed mutagenesis using *cagA-1CM*^*W2*^ as template. The mutagenized genes were inserted into pCDH-EF1 to make pCDH-EF1-CagA-1CM^W2^ and pCDH-EF1-CagA-1CM^E^. These pCDH-EF1-CagA vectors were used to generate pseudoviral particles based on the Lentiviral Expression System (System Biosciences).

### AGS culture and transfection

AGS human gastric epithelial cells were cultured in RPMI 1640 medium supplemented with 10% fetal bovine serum at 37 °C in 5% CO_2_. For microscopy, AGS cells were seeded in 8-well chamber wells at 0.2 × 10^5^ cells/well and after 12 h, transfected with 0.24 μg of pCDH plasmids using Lipofectamine 2000 (Invitrogen). Cells were fixed for stress fiber analysis 24 h after transfection.

### Immunoblotting

Preparation of AGS total cell lysates and immunoblotting were performed as described previously^46^.

### Immunostaining and microscopy

AGS cells were fixed and permeabilized as described previously^12^. Cells were treated with primary antibodies followed by visualization with Alexa Fluor-conjugated secondary antibodies (Invitrogen). F-actin was stained with Alexa Fluor 488-conjugated phalloidin (Invitrogen) and nuclei were stained with DAPI (Wako). The focal plane of the confocal microscope was set at the cell bottom. Images were acquired using an Olympus FV1200 laser confocal microscope. Images were analysed using the Image J software (NIH)^58^.

### Quantification and statistics for stress fiber assay

AGS cells, which displayed at least one stress fiber that transected the middle 50% of the cell body or the nucleus, were considered stress fiber-positive. The percentage of cells with stress fiber (mean ± range %) was calculated from three independent experiments. The repeated measures one-way ANOVA and post-hoc Tukey’s test were performed using the GraphPad Prism 6 software (GraphPad Software) based on an assumption that the data are normally distributed. Alpha level was set at 0.05 (two-tailed).

### Transepithelial electrical resistance (TER)

MDCK cells were allowed to form polarized monolayers for three days on transwell filters (#3460, Corning) and then infected with pseudoviral particles from the basal side at 200 MOI (multiplicity of infection) for 24 hours in the presence of 8 μg/ml polybrene. Resistance of samples were measured using Millicell ERS-2 (Millipore) and blank values were subtracted off to calculate the resistance of cell monolayers.

## Additional Information

**How to cite this article**: Nishikawa, H. *et al*. Impact of structural polymorphism for the *Helicobacter pylori* CagA oncoprotein on binding to polarity-regulating kinase PAR1b. *Sci. Rep.* **6**, 30031; doi: 10.1038/srep30031 (2016).

## Supplementary Material

Supplementary Information

## Figures and Tables

**Table 1:** Summary table of binding affinities between various CagA constructs and PAR1b.

GST-CagA constructs	Type of CM motif	CM sequence	No. of CM motifs	PAR1b binding	K_D_ ± SE (nM)
∆CM	—	—	0	N	ND
1CM^W1^	Western	FPLKRHDKVDDLSKVG	1	Y	90.3 ± 14.1
2CM^W^	Western	FPLKRHDKVDDLSKVG	2	Y	26.6 ± 3.7
4CM^W^	Western	FPLKRHDKVDDLSKVG	4	Y	2.43 ± 0.45
1CM^W2^	Western	FPLKRHDKVDDLSKVG	1	Y	106 ± 19
1CM^E^	East Asian	FPLRRSAAVNDLSKVG	1	Y	46.4 ± 7.4
1CM^AmI^	Amerindian I	SSLKRHAKVDDLSKVG	1	N	ND
1CM^AmII^	Amerindian II	YTLKMHAGDDNLRSKVG	1	N	ND

ND: not determined.
