# Supplementary material for: Impact of structural polymorphism for the Helicobacter pylori CagA oncoprotein on binding to polarity-regulating kinase PAR1b
Source: Sci Rep. 2016 Jul 22;6:30031. doi: 10.1038/srep30031 (PMC4957108; doi:10.1038/srep30031)
Supplement: Supplementary Information [file srep30031-s1.pdf]

Supplementary Information

Impact of structural polymorphism for the *Helicobacter pylori* CagA oncoprotein on binding to polarity-regulating kinase PAR1b

Hiroko Nishikawa, Takeru Hayashi, Fumio Arisaka, Toshiya Senda & Masanori Hatakeyama

Supplementary Methods

Bacterial expression vector for full-length PAR1b

Human *PAR1b* (GenBank: NM_017490.3) was tagged at the 3' end with the 6xHis tag sequence by PCR and inserted into pGEX-6P-2 (GE Healthcare).

Full-length PAR1b expression and purification

Protein expression and purification were performed as previously described[1] with a few modifications. High salt lysis buffer (PBS, 2% (v/v) Tween 20, 1% (v/v) Triton X-100, 1 mM EDTA, 1 mM DTT, 350 mM NaCl) was used for the initial lysis of cells. Cleared lysate was mixed with Ni Sepharose excel beads (GE Healthcare) and eluted in high salt lysis buffer + 500 mM imidazole, prior to the GST purification step. The final incubation with PreScission Protease (GE Healthcare) was performed in 50 mM Tris-Cl, pH7.5, 500 mM NaCl, 1 mM EGTA, 1 mM DTT.

Supplementary Reference

1. Kojima, Y. et al. Suppression of tubulin polymerization by the LKB1-microtubule-associated protein/microtubule affinity-regulating kinase signaling. *J. Biol. Chem.* **282,** 23532–23540 (2007).

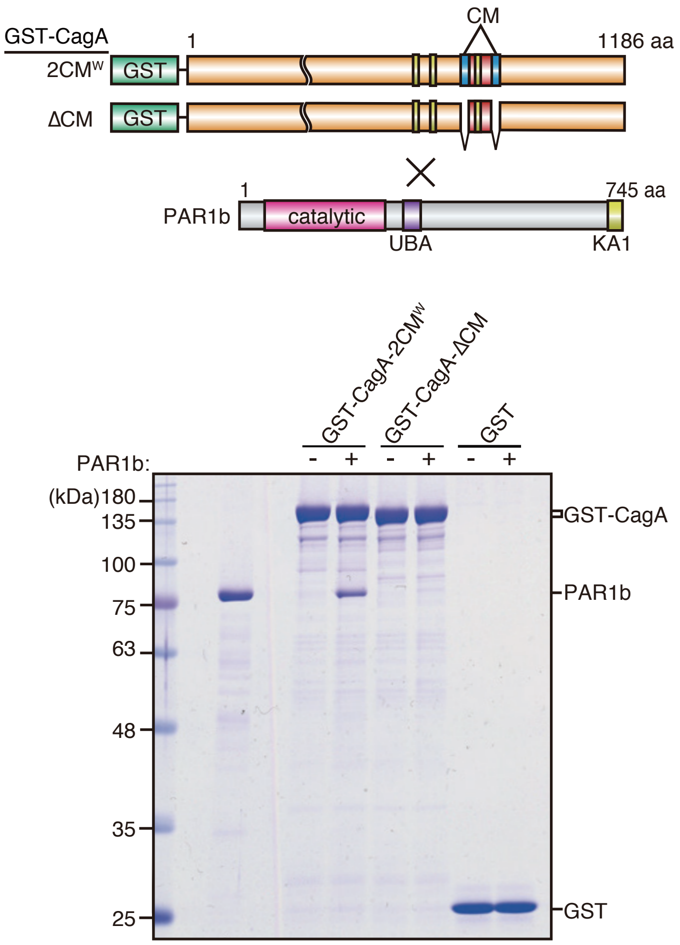

Supplementary Figure S1. CM-dependent interaction between CagA and full-length PAR1b. Schematic diagram of GST-CagA-2CM^{W}, GST-CagA-ΔCM and PAR1b used in this pull-down assay *(top)*. GST pull-down was performed in GST binding buffer + 0.01 % Triton X-100. Samples were resolved on an SDS-PAGE gel and stained with CBB *(bottom)*.

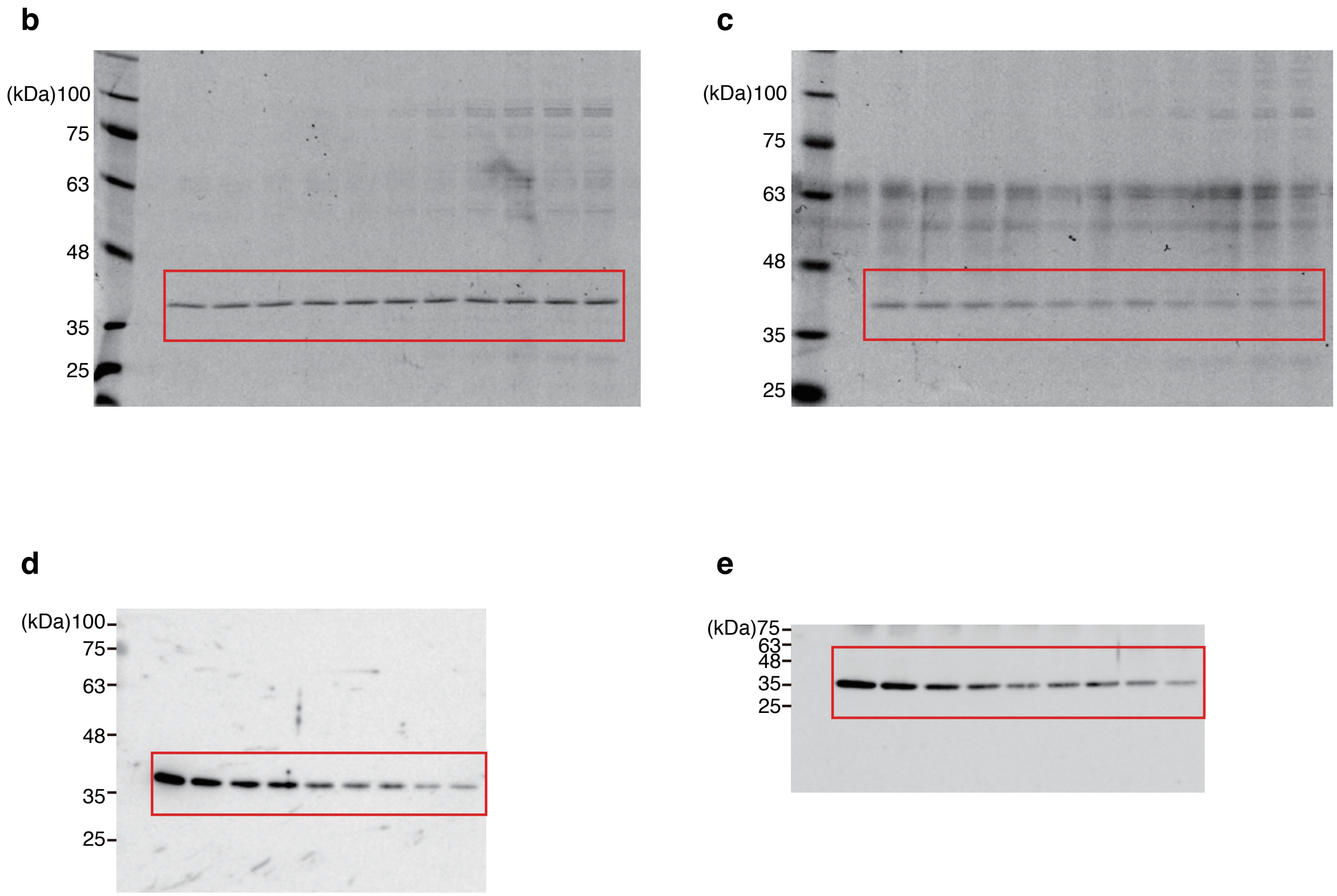

Supplementary Figure S2. Full-length gels and blots for Figure 3b-e. (b&c) Full-length CBB stained gels. **(d&e)** Full-length immunoblots detected by α-FLAG antibody. Red squares indicate where the images were cropped.

b

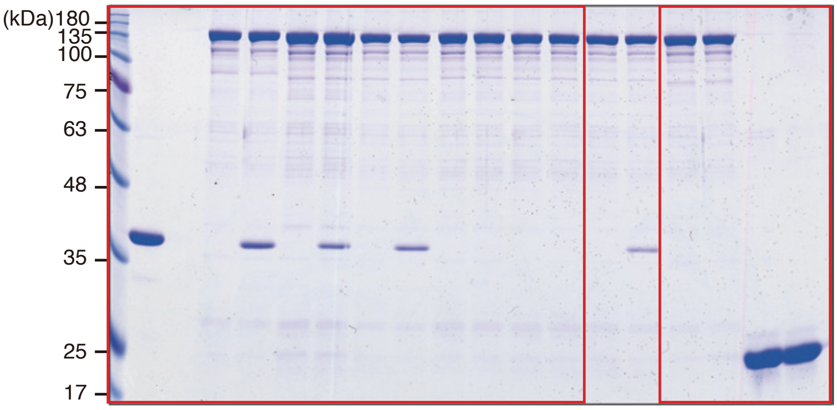

c

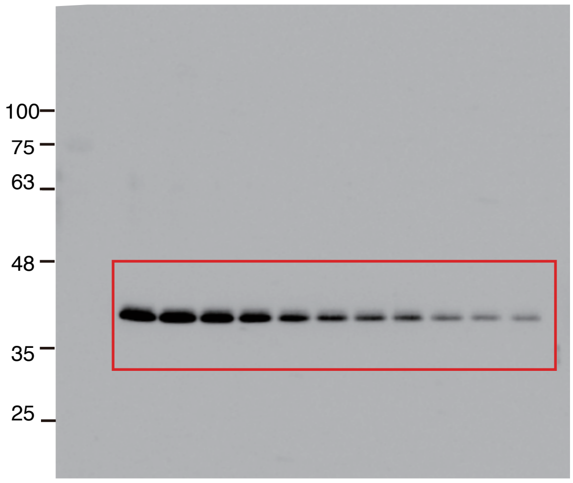

d

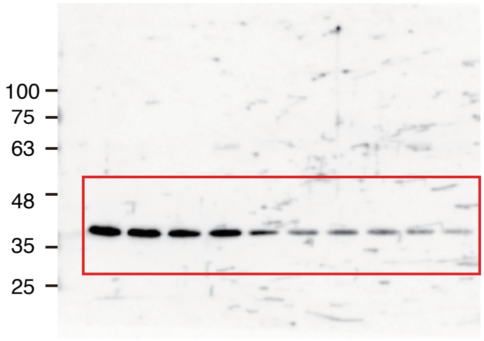

Supplementary Figure S3. Full-length gels and blots for Figure 4b-d. (b) Full-length CBB stained gel. **(c&d)** Full-length immunoblots detected by α-FLAG antibody. Red boxes indicate where the images were cropped.

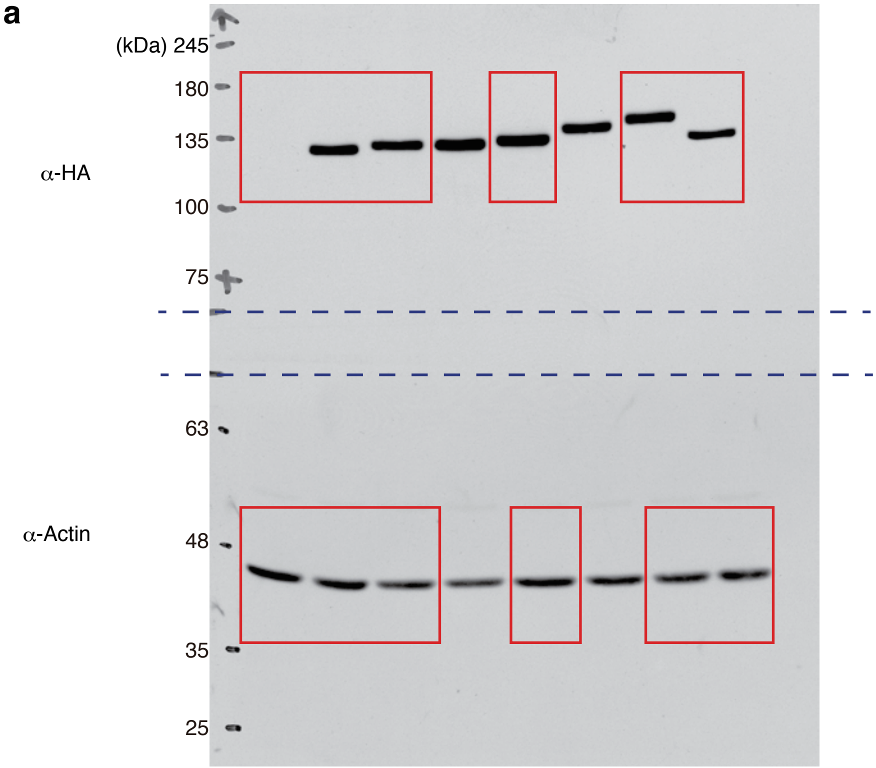

Supplementary Figure S4. Full-length blot for Figure 5a. Blue dashed lines indicate where the membrane was cut and probed with different antibodies. Red squares indicate where the images were cropped.

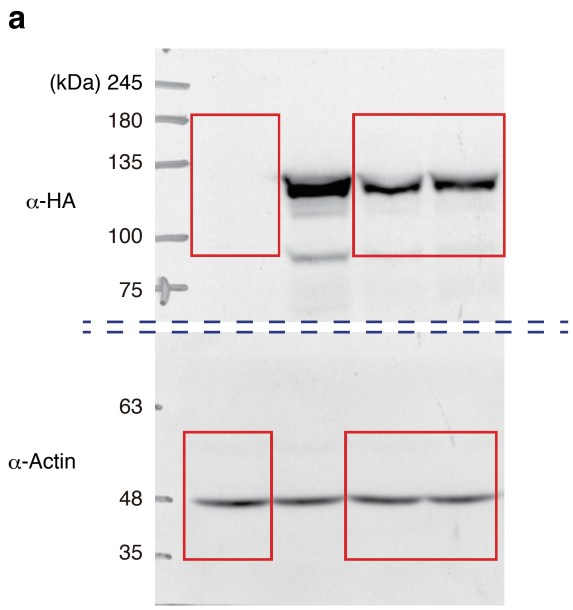

Supplementary Figure S5. Full-length blot for Figure 6a. Blue dashed lines indicate where the membrane was cut and probed with different antibodies. Red squares indicate where the images were cropped.
